# Hosting the Tobacco Industry Supply Chain and Political Interference

**DOI:** 10.1093/ntr/ntad178

**Published:** 2023-09-14

**Authors:** Rosemary Hiscock, Hala Alaouie, Britta K Matthes, John Mehegan, Michael J Bloomfield

**Affiliations:** Tobacco Control Research Group, Department for Health, University of Bath, Bath, UK; Tobacco Control Research Group, Department for Health, University of Bath, Bath, UK; Department of Social and Policy Sciences, University of Bath, Bath, UK; Tobacco Control Research Group, Department for Health, University of Bath, Bath, UK; Centre for Development Studies, University of Bath, Bath, UK; Tobacco Control Research Group, Department for Health, University of Bath, Bath, UK; Tobacco Control Research Group, Department for Health, University of Bath, Bath, UK; Department of Social and Policy Sciences, University of Bath, Bath, UK; Centre for Development Studies, University of Bath, Bath, UK

## Abstract

**Introduction:**

We found no comprehensive studies on the location of transnational tobacco and leaf company (TTLC) subsidiaries (business entities they control) or the consequences of their presence on health policy.

**Aims and Methods:**

Here we assess (1) the global reach of TTLCs by mapping their tobacco growing and manufacturing subsidiaries and (2) the relationship between in-country presence of the tobacco industry and their power and interference. Data on subsidiaries were collated through systematic searching for countries’ supply chain activities in documents and web pages. Cross-sectional multiple regression analysis was used to assess the association between the number of agricultural and manufacturing TTLCs and the Tobacco Industry Interference score, and the degree to which these were mediated by tobacco control, good governance, and economic importance of tobacco.

**Results:**

TTLC supply chain activity had global reach. As the number of TTLCs with tobacco growing and manufacturing activities rose, interference increased significantly. Interference was associated with poorer tobacco control. The association with more TTLCs undertaking final product manufacturing was related to higher-value tobacco exports but was not related to tobacco making a bigger contribution to the economy.

**Conclusions:**

TTLCs continue to control the global tobacco supply chain through their globe-spanning subsidiaries. The presence of TTLCs in a country is associated with political interference. Countries should consider their participation in the tobacco supply chain alongside the understanding that they are likely to cede political power to TTLCs, potentially undermining the health of their populations.

**Implications:**

Tobacco control research has traditionally concentrated on the demand side of tobacco. Our results lend support to calls for more research on the supply of tobacco. Governments should require tobacco companies to provide detailed, up-to-date information in an easily accessible format on in-country supply chain activities. Policymakers should take the likelihood of political interference in health and environmental policy into account when making decisions about foreign direct investment offered by the tobacco industry.

## Introduction

Tobacco kills half of smokers.^1^ Despite this, the number of smokers continues to grow^2^ and the tobacco industry remains one of the most profitable industries in the world.^3^ Like other global industries, the tobacco industry’s significant finances, their promise of investment, employment, and tax revenue opportunities, and the prevailing discourse of economic priorities trumping public health concerns give them significant power to shape public policy in the countries that host them.^4,5^ For example, such power enables tobacco companies to grow their profits in part by interfering directly or indirectly with government implementation of internationally agreed tobacco control measures (the World Health Organization Framework Convention on Tobacco Control [WHO FCTC]) with the goal of preventing, weakening, delaying, and undermining them.^6^ Interference is a key barrier to tobacco control progress.^6^ Thus, it is important to address industry interference to strengthen tobacco control.

In 2015, the world market for tobacco was controlled by four cigarette companies: Philip Morris International, British American Tobacco, Japan Tobacco International, and Imperial Brands (IMB).^7^ These four companies continued to dominate the cigarette market of most countries in 2020.^8^ Current statistics suggest they are elevated among tobacco companies on various common measures of company size (Table 1). Given their global operations, they are known as transnational tobacco companies (TTCs). Other players are geographically limited (Altria [USA], China National Tobacco Company [China], and ITC [India]) or are chiefly important for minor tobacco product markets such as cigars and cigarillos, for example Swisher International.

**Table 1.** Transnational Tobacco Companies (TTCs) Ranked by Common Measures of Company Size in 2020, 1 Being the Highest Rank^1^

TTC	Cigarette volume share	Cigar and cigarillo volume share	Smoking tobacco^2^ volume share	Market value	Net sales	Average rank
* Source*	*Euromonitor* ^3^	*Euromonitor*	*Euromonitor*	*Forbes* ^4^	*Forbes*	
BAT	2	5	3	3	1	2.8
JTI	4	1	1	5	5	3.2
IMB	5	3	2	6	3	3.8
PMI	3	11	4	1	2	4.2
Volume (unit)^5^	5 trillion	43 billion	230 billion			
Value US$	717 billion	42 billion	34 billion			

^1^Other companies that ranked higher than at least one of the TTCs were Altria (on three measures), China National Tobacco Company (CNTC) (on two measures but note CNTC did not appear on Forbes list) and on one measure: Swisher International, Scandinavian Tobacco Company, PT Inter Tobacco Utama, The Burger Group, Swedish Match and ITC.

^2^Smoking tobacco is used for Roll Your Own and Make Your Own cigarettes, pipes and waterpipes (shisha).

^3^Euromonitor International data was downloaded on January 6, 2022. Note Euromonitor has received money from the tobacco industry^9^.

^4^Forbes tobacco company data were collated by Statista^10,11^.

^5^1 unit = 1 cigarette stick, 1 unit, 1 gram, respectively.

Furthermore, two global tobacco leaf suppliers, Pyxus (formerly Alliance One) and Universal Corp, also exerted significant control over the world tobacco market in 2015.^7^ At the end of 2020, Fitch Ratings (the credit rating agency) described Universal and Pyxus as “the only two global tobacco leaf suppliers that operate in all key regions for tobacco production” with other distributors being local or regional and lacking the infrastructure to expand successfully.^12^

Together these six companies can be labeled as transnational rather than multinational because their foreign-based subsidiaries have some decision-making powers and can adapt their activities to local needs.^13^ In this article, they will be collectively referred to as Transnational Tobacco and Leaf Companies (TTLCs). Each of these TTLCs has numerous “subsidiaries.” A subsidiary is a company controlled by another company (known as the “parent” company). Control is attained often via the parent owning more than 50% of a subsidiary’s shares.^14^

Tobacco control advocates and localized studies suggest tobacco industry interference with policymaking can be particularly resonant in countries where there is employment associated with tobacco growing and manufacturing.^15–18^ transnational tobacco and leaf company (TTLC) tobacco leaf growing subsidiaries operate increasingly via contracts between the company and farmers.^19^ Manufacturing involves primary processing facilities (processing tobacco leaf) and secondary processing factories (manufacturing final tobacco products). Such subsidiaries are part of the tobacco supply chain which consists of the processes, actors, and supporting industries involved in bringing tobacco from the field to the smoker.^20^

Mapping the supply chains of corporations with a global reach has been recommended to better understand where public health, equality, and environmental policy may be undermined because of corporate activity, and where workers and the environment may be exploited to increase profits.^21^ Such mapping could be used to draw attention to the potential tradeoffs involved in hosting tobacco supply chain activities while also garnering support for governments and public health leaders in low- and middle-income countries (LMICs). LMICs have been found to be especially vulnerable to tobacco industry interference due to a lack of resources and political will,^22^ often accompanied by the tobacco industry’s better reputation than in high-income countries, and more direct access to politicians in countries with less oversight of industry-government relations, expanded control over the media and lax marketing guidelines.^17^ Mapping could facilitate the development and implementation of measures that (better) protect policies from commercial and other vested interests of the tobacco industry,^6^ the latter of which include independent companies that supply inputs such as chemicals or packaging.^20,23^

Although there have been studies on the tobacco supply chain in specific areas, the global footprint of TTLC subsidiaries has yet to be mapped.^20^ This means the global tobacco control community lacks an overarching picture of where subsidiaries are located and are, therefore, likely to exert influence over public policy, and an understanding of why they occur in particular patterns. Accordingly, the objectives of this article are to map the geographical presence of the TTLCs and their tobacco supply chain activities associated with growing and manufacturing, and to take a first step toward better understanding the extent to which in-country activity is associated with political interference, good governance, tobacco control development, and economic tradeoffs.

## Methodology

This study consisted of mapping supply chain activities and cross-sectional analysis of supply chain activity and tobacco industry interference and potential confounding political and economic indicators.

### Data

Data on TTLC subsidiaries were collated for the Tobacco Supply Chains Database.^23^ Prior to data collection, we explored the tobacco supply chain identifying the journey of tobacco (from seed to ash), process steps, and actors.^20^ TTLC subsidiaries and their activities were identified by systematically searching TTLC annual reports, sustainability reports, and, where available, government filings and TTLC webpages (particularly subsidiary webpages and sustainability webpages). Internet searches included, for example, TTLC name (in full or part and including subsidiary name if applicable) and location (country and sometimes address) and supply chain activity. Local language terms were used if appropriate reducing the bias towards companies operating in English-speaking countries. Searches took place in 2021 and the data was made available for analysis on November 17, 2021. For every country, we identified whether there was evidence of TTLCs undertaking tobacco growing, leaf processing, and manufacturing final tobacco products and calculated the number of TTLCs undertaking each of these activities.

Previous research has suggested that a lack of financial resources at a national level may have consequences for political interference,^17,22^ thus country income grouping based on gross domestic product per capita in 2020 (2021 data were not available at the time of download)^24^ was included as a control variable. Low-income and lower-middle-income countries were merged for sample size reasons.

The Global Tobacco Industry Interference Index (TIII) assesses: level of industry participation in policy development, tobacco-related corporate social responsibility activities, benefits to the tobacco industry, forms of unnecessary interaction with tobacco industry, transparency, conflicts of interest, and preventive measures. The TIII was established to review government responses to tobacco industry interference, complementing government self-reporting on the implementation of Article 5.3 WHO FCTC which requires preventing the tobacco industry or vested interests from participating in health policy development. In 2021, eighty countries participated and were scored from low (countries that have strong measures in place to protect against tobacco industry interference) to high (countries that have weaker measures in place to protect against tobacco industry interference); country’s scores ranged between 15 (Brunei Darussalam) and 96 (Dominican Republic).^25^ The TIII was the main outcome measure in the study.

Three potential mediators of any association between TTLC subsidiaries and industry interference were analyzed: tobacco control, good governance, and tobacco’s contribution to the economy. These mediators encompass concepts that can shape the ability of industry to influence the political environment including regulatory framework, institutions, and economics.

Countries’ tobacco control measures were assessed using MPOWER 2019—the latest available at the time of download.^26^ The MPOWER score measures the adoption of key WHO FCTC provisions in each member state. High scores indicated effective tobacco control policies, which align with the WHO FCTC. Given incomplete reporting and to maximize the number of countries in the analysis, analysis was restricted to the MPOWER index (*n* = 195 countries) and the following three out of six subscales: Monitoring prevalence, Warnings on packaging, and Enforcing advertising bans.

Strength of public governance was measured via the 2021 World Governance Indicators: voice and accountability ­(citizens' participation in choice of government and freedom of expression and association), political stability and absence of violence or terrorism, government effectiveness (quality of civil service and its independence), regulatory quality (government ability to regulate and promote private sector development), rule of law (confidence in an abiding by society’s rules), control of corruption (extent government is controlled by private interests or operates for private gain).^27^ The indicators are gathered from a wide variety of sources under the auspices of the World Bank. Indicators are provided as an estimate and as a percentile rank among all countries. High scores indicate good governance. Data for the six indicators were available for between 208 and 213 jurisdictions.

Contribution of tobacco to the economy was measured by the absolute value of tobacco exports in U.S. dollars and the percentage of total export value tobacco accounted for, both downloaded from the United Nations Comtrade Database.^28^ Because few countries had reported for 2021 at the time of download, 2020 data (*n* = 377 countries and territories) was used, except for countries without 2020 data. For such countries we used the most recent data available: Ghana (2019), Iran, Sudan, and Solomon Islands (2018), Iraq (2016), Bangladesh and Brunei Darussalam (2015). Tobacco was defined as the World Customs harmonized commodity code HS24.^29^ From 2015 to 2020 this code included tobacco leaf and tobacco leaf refuse; conventional tobacco products such as cigarettes, cigars, and smoking tobacco; chewing tobacco and snuff; tobacco for heated tobacco products, tobacco extracts, and essences for pesticides, but did not, during this period, include e-cigarettes and nicotine replacement therapy.^30–32^

### Analysis

#### Mapping of Subsidiaries

Maps showing the number of TTLCs active in each country by supply chain process (tobacco growing, leaf processing, and final product manufacture) were created using Microsoft Excel. There were no exclusions.

#### Association Between Subsidiaries and Interference

There were 76 countries included in the analysis. Tobacco export data were not available for four of the 80 countries with a TIII score and these were excluded (Gabon, Palau, Maldives, and Venezuela). Primary processing was not included in the statistical analysis because primary processing subsidiary locations were very similar to agriculture subsidiaries. There were two subsidiary variables analyzed: The number of TTLCs growing tobacco in the country and the number of TTCs manufacturing final products in the country. Countries with two or more TTLCs growing tobacco were merged for analysis as were countries with three or more TTCs manufacturing final products.

Bivariable associations were assessed using nonparametric statistics between tobacco control, governance, and export contribution and TIII (Spearman’s Rho) and the TTLC subsidiaries (Kruskall–Wallis) due to non-normal distributions ([Kolmogorov Smirnov and Shapiro Wilks *p* < .05] see Supplementary Table S1).

The TIII met the assumptions for a multivariable linear regression outcome variable. It was normally distributed and displayed homogenous variance (see Supplementary Table S1 and Box S1). Linear regression modeling was used for multivariable analysis using SPSS version 28.000 GENLIN command.

In multivariable modeling, the outcome variable was the TIII and the dependent variables of most interest were the number of TTLCs growing tobacco and the number of TTLCs manufacturing final products. In the basic model, an association between TTLC subsidiaries (growing and manufacturing) and TIII was established after taking into account country income. A sensitivity analysis excluding an outlier country confirmed the results (see Supplementary File). In the subsequent eight models, the eight indicators associated with either the TIII or TTLC subsidiaries (growing or manufacturing) *p* < .10 in bivariable analysis, were entered singly into the basic model, and the change in the association between TTLC subsidiaries (growing and manufacturing) and TIII was assessed.

We have reported all measures, conditions, and data exclusions, as well as how sample sizes were determined.

## Results

### Mapping Global Supply Chain Activity of the TTLCs

**Figure 1.**
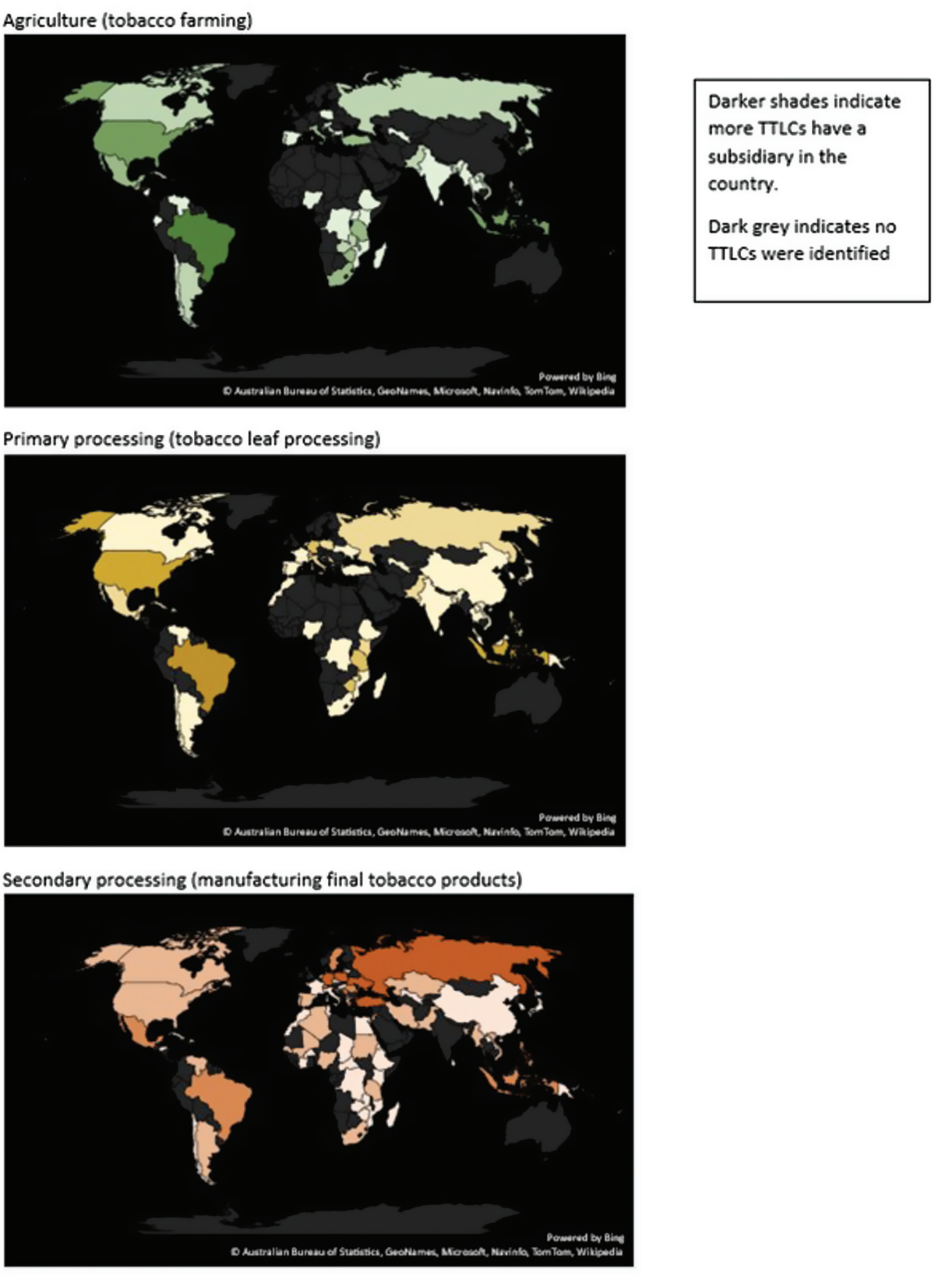
Location of subsidiaries undertaking agricultural, primary processing or secondary processing activities.

We found 47 countries with TTLC subsidiaries carrying out agricultural activities (growing tobacco), 51 primary processing (processing tobacco leaves), and 74 secondary processing (manufacturing tobacco products). As expected, the TTCs (British American Tobacco, IMB, Japan Tobacco International, and Philip Morris International) undertook secondary processing but the two leaf transnationals (Universal and Pyxus) did not (Table 2). Final product manufacture subsidiaries were more widespread in Europe and Northern Africa than tobacco growing subsidiaries (Figure 1).

**Table 2.** Countries With Transnational Tobacco and Leaf Company Supply Chain Processes by Company

Supply chain process:	*N* countries	BAT	IMB	JTI	PMI	Pyxus	Universal
Agriculture (growing tobacco)	47	23	2	11	13	16	18
Primary processing (of the leaf)	51	25	6	11	11	13	16
Secondary processing (manufacturing final tobacco products)	74	44	25	30	31	0	0

PMI = Philip Morris International, BAT = British American Tobacco, JTI = Japan Tobacco International,

### Supply Chain Activity’s Political and Economic Bivariable Associations

Bivariable analysis (Supplementary Table S2) revealed a statistically significant association between the number of TTLC subsidiaries in a country and tobacco industry interference. For both, agricultural subsidiaries and final product manufacturing subsidiaries, countries with the highest number of TTLC subsidiaries had significantly higher industry interference (*p* < .05).

Interference was also associated with less effective tobacco control overall, less effective advert bans (*p* < .05) and perhaps less effective pack warnings (*p* < .10). The association between monitoring smoking prevalence and final product subsidiaries also approached significance (*p* < .10).

Good governance was not significantly associated with tobacco industry subsidiaries or industry interference. However, the association between better regulatory quality (more effective regulation of private sector development) and fewer agricultural subsidiaries approached significance (*p* < .10).

All associations with tobacco exports were significant (*p* < .05). Higher tobacco exports and a greater contribution of tobacco to overall exports were associated with higher levels of industry interference and the presence of more TTLC agricultural and final product subsidiaries. Countries with no agricultural subsidiaries reported median tobacco exports of US$39 million whereas countries with two or more agricultural subsidiaries reported median tobacco exports of US$206 million, comprising a median of 0.1% and 0.4% of the value of total exports, respectively. Countries with no final product subsidiaries reported median tobacco exports of US$18 million whereas countries with three or more final product subsidiaries reported median tobacco exports of US$1030 million, comprising a median of 0.1% and 0.7% of total exports, respectively.

### Supply Chain Activity and Tobacco Industry Interference—the Basic Multivariable Model

In multivariable regression modeling, model fit, as measured by log-likelihood, was optimized in a model including country income, number of TTCs involved in secondary processing, and number of TTLCs growing tobacco (Supplementary Table S2). In this basic model (see Table 3), countries with one TTLC growing tobacco were 12 points lower on the interference scale (*p* = .014) and countries with no growing were 7 points lower (*p* = .085). Countries with no final product manufacturing had scores 13 points lower on the interference scale than countries with two or more TTCs manufacturing (*p* = .010).

**Table 3.** Linear Regression Modeling of the Association Between Transnational Tobacco and Leaf Company Subsidiaries and Interference and the Impact of Tobacco Control, Governance and Tobacco’s Economic Contribution

New variable entered:	Basic model			MPOWER			Monitor prevalence			Warn on pack			Enforce advert ban		
	B	SE	p	B	SE	p	B	SE	p	B	SE	p	B	SE	p
*Country income*			<.001			<.001			<.001			.001			<.001
Lower middle (&low) income	3.0	3.6	.412	-0.5	3.5	.883	1.7	3.9	.663	2.3	3.5	.518	4.7	3.5	.181
Upper middle income	15.0	3.8	<.001	12.6	3.6	.001	14.0	4.1	.001	13.2	3.8	<.001	16.2	3.7	<.001
High income*	0			0			0			0			0		
*N TTLC undertaking growing*			**.046**			.023			.043			.075			.181
no TTLCs	-6.8	4.0	.085	-7.4	3.7	.047	-6.9	4.0	.083	-6.1	3.9	.115	-4.7	3.9	.223
1 TTLC	-11.5	4.7	.014	-11.8	4.4	.007	-11.7	4.7	.012	-10.3	4.6	.023	-8.5	4.6	.065
>1 TTLCs *	0			0			0			0			0		
*N TTC final product making*			**.008**			.009			.008			.004			.009
no TTCs	-12.7	4.9	.010	-13.3	4.6	.004	-13.3	4.9	.007	-14.5	4.8	.003	-12.2	4.7	.009
1 TTC	-0.5	5.2	.923	-3.3	4.9	.501	-1.6	5.4	.764	-3.4	5.2	.515	-1.1	4.9	.818
2 TTCs	-5.2	4.9	.285	-7.3	4.6	.114	-5.9	4.9	.235	-7.6	4.8	.115	-4.0	4.7	.385
>2 TTCs *	0			0			0			0			0		
New variable				-4.4	1.3	.001	-1.3	1.8	.456	-3.2	1.4	.024	-4.4	1.5	.004
Model Fit (Log Likelihood)	-299.087			-293.760			-298.810			-296.633			-295.247		
New variable entered:	**Regulatory quality (est)**			**Regulatory quality (rank)**						**Tobacco export value**			**% exports tobacco**		
*Country income*	<.001			<.001						<.001			<.001		
Lower middle (&low) income	2.8	7.1	.693	0.9	6.7	.888				5.1	3.9	.194	1.5	3.5	.676
Upper middle income	14.9	5.8	.010	13.7	5.4	.011				16.6	4.0	<.001	13.3	3.8	<.001
High income*	0			0						0			0		
*N TTLC undertaking growing*			.048			.043						.026			.011
no TTLCs	-6.8	4.0	.086	-6.8	4.0	.088				-7.1	3.9	.071	-7.9	3.8	.040
1 TTLC	-11.6	4.7	.014	-11.8	4.7	.013				-12.7	4.7	.007	-13.7	4.6	.003
>1 TTLCs *	0			0						0			0		
*N TTC final product making*			.008			.007						.042			.019
no TTCs	-12.7	4.9	.010	-13.0	5.0	.009				-7.9	6.1	.199	-11.1	4.7	.019
1 TTC	-.5	5.3	.920	-0.8	5.3	.874				3.5	6.0	.555	-0.2	5.0	.967
2 TTCs	-5.3	5.0	.297	-5.7	5.0	.261				-1.4	5.7	.809	-5.2	4.7	.265
>2 TTCs *	0			0						0			0		
New variable	-.1	3.4	.977	0.0	0.1	.725				0.0	0.0	.199	2.5	1.0	.013
Model Fit (Log Likelihood)	-299.086			-299.025						-298.270			-296.116		

^*^Reference category: In multivariable modeling, interference scores of the other within-variable groupings are compared to the reference category.

Income (Gross National Income (GNI) per capita in 2021 U.S. dollars) boundaries: High income > US$12695, upper middle income US$4096 to US$12695, lower middle (& low income) < US$4096^33^.

Interference was 15 points higher in upper-middle-income countries than in high-income countries (*p* < .001). Interference in low- and lower-middle-income countries was similar to high-income countries (*p* = .412).

### What Impacts the Relationship Between Supply Chain Activity and Tobacco Industry Interference?

The association between TTLC subsidiaries and industry interference in the base model was compared to models with other explanatory variables entered singly (Table 3). The significant association between the number of TTLCs growing tobacco and interference only disappeared (*p* = .181) in one model: The model where enforcing advert bans was entered. Better enforcement of advert bans was significantly associated with less interference (*p *< .004). The model fit (log-likelihood) improved from −299 to −295.

In the model where absolute export value (US$) was entered, the significant advantage of having no tobacco manufacturing for interference compared with having three or more TTCs manufacturing disappeared (*p* = .199). Absolute export value did not itself reach significance (*p* = .199) and the model fit hardly changed (−298) implying that absolute export value and number of TTCs manufacturing final products are more or less interchangeable statistically. The other potential mediators did not change the association between final product manufacture and industry interference.

In these multivariable models, poorer tobacco control—as indicated by the MPOWER index (*p* = .001), weaker packaging warnings (*p* = .024), and weaker enforcement of advert bans (*p* = .004)—continued to be associated with more interference. Higher reliance on tobacco in the economy in terms of proportion of exports from tobacco also continued to be associated with more interference (*p* = .013).

## Discussion

TTLCs grow tobacco in countries of all income groups, from the United States to Zambia, and in countries from all continents (except Antarctica). Universal explains that a “global presence allows us to meet our customers’ diverse product requirements while minimizing the effects of adverse crop conditions and other localized supply disruptions.”^34^ Thus, having subsidiaries distributed around the world is advantageous to TTLCs and reflects a deliberate strategy to ensure a reliable supply of tobacco leaves.

Manufacturing is even more widespread globally. Although it might be thought that LMICs would, in essence, be predominantly a farming resource for TTLCs, we found that there is also extensive secondary processing (final product manufacturing) in LMICs. Tobacco product manufacturing has sometimes been described as “value added” (final products generate higher prices than tobacco leaf) and a route to development.^35^ In simple supply or value chain terms, presence of these subsidiaries could indeed be seen as a success with LMICs participating in these higher value-added supply chain activities. However, the location of growing and manufacturing has consequences: Our study suggests that hosting the tobacco industry, especially in countries where several of the TTLCs operate, is associated with a higher level of interference in tobacco control policymaking. In particular, we found that countries where a higher number of TTLCs conducted tobacco farming had weaker enforcement of advertising bans. Comprehensive advertising bans reduce smoking uptake and increase quitting; advertising bans have been described as a cost-effective best buy and a cornerstone of tobacco control policy.^36,37^

How might the presence of TTLC activities lead to interference? Where growing or manufacturing takes place, industry-linked people could enter government and set up public bodies (tobacco boards) to promote tobacco which aids in forming links with decision-makers, and otherwise make now standard industry arguments around, for example, how farmers would find it difficult to diversify to other crops, and that stopping tobacco growing would reduce access to food and education.^17^ These decision-makers may sit in the trade, export, and finance ministries which may be less stringent in applying WHO FCTC measures than health ministries. In addition, TTLCs increasingly engage farmers through contracts where a TTLC is the sole buyer, which could facilitate the mobilization of farmers against tobacco control measures.^38^ Furthermore, the presence of a subsidiary in a country allows companies to engage in locally relevant corporate social responsibility activities, giving them a greater presence and potentially enhanced legitimacy in the public domain^18^ despite having been shown to predominantly favor only TTLC profitability.^39^ Such discursive and instrumental strategies serve to prevent, delay, or weaken tobacco control policies and hence pose a threat to public health.^40^

Data from this study also confirms previous findings^19^ that tobacco makes only a meager contribution to economies, even in those countries with the most TTC manufacturing subsidiaries (0.7%). The association between the number of TTLCs with a subsidiary in a country and interference was not impacted by the contribution of tobacco to the economy in percentage terms but by its contribution in monetary value. This seems to indicate that TTLCs are more likely to interfere in economies where they are creating higher value from tobacco. Governments should not be overawed by absolute value of exports generated, particularly because much of that value will be extracted as profits to private corporations often headquartered elsewhere^3,41^ rather than used to benefit the local population^19^ and comes with significant hampering of tobacco control development at home. Forty years of an economic paradigm of balancing public and environmental health against the freedom and expansion of unhealthy commodity industries have left a legacy of inequality and environmental harm.^41^ Controlling the supply side of tobacco, in addition to the demand side (thus far the main focus of tobacco control), is vital.^42^

### Limitations

First, neither official reporting (annual and financial reports) nor company websites bore comprehensive lists of company subsidiaries. Even U.S. Securities may miss subsidiaries^43^ which could be responsible for substantial public health and environmental damage. Information for some TTLCs, subsidiaries, and countries was more available and transparent, depending on reporting requirements, media focus, and press releases.

Second, the subsidiaries' data were extracted prior to the Russian invasion of Ukraine in March 2022. The war has disrupted tobacco industry activity in Ukraine and the TTCs have stated they are reducing their activities in Russia.^44^

Third, we restricted our search to supply chain activities for traditional tobacco products such as cigarettes, cigars, roll-your-own tobacco, waterpipe, and Snus which have long been present, and their harms confirmed. TTLCs are, however, at least claiming to be pivoting towards newer nicotine and tobacco products, particularly heated tobacco products; the harm caused by heated tobacco products is currently unclear and most evidence is not independent of the tobacco industry,^45^ and so these products were not included. We restricted our analysis to growing and manufacturing subsidiaries given their association with employment. Some countries with no manufacturing or growing subsidiaries nevertheless exported tobacco (see Supplementary Table S3). Future studies could make more use of trade data to understand the importance of subsidiaries with a logistical focus.

Fourth, the analysis was hampered by the availability of measures of potential mediators. There are other concepts that could be included, such as each country’s tax and revenue structure, political geography (the spatial dimensions of politics), and the spread of the tobacco supply chain within countries. Furthermore, case studies could be undertaken to explore reasons for the patterning of TTLC’s activities, such as labor costs, raw resources as well as the presence of trading hubs. In addition, TIII data were only from 76 countries and only three of the MPOWER subscales could be included. To avoid losing more countries from the analysis, we only used tobacco exports as an indicator of economic contribution and did not include domestic sales data. The analysis was cross-sectional, so the direction of causality of associations between presence of TTLCs and TIIII is uncertain. In future, the tobacco supply chain database can be a mechanism for ongoing tracking of the location of subsidiaries.^23^

Fifth, although we used leading scales supported by leading international organizations, they are not exempt from criticism. MPOWER score represents tobacco control legislation but not implementation or compliance.^46^ The TIII measures agency power rather than structural power (where governments act in the interests of industry because of the perceived economic benefits even without TTLCs prompting). However previous work suggests that such structural power is not infinite, so TTLCs often engage in more direct interference activities as well and the two dimensions of power reinforce each other.^47^ Recent criticisms from India of the World Governance Indicators have been largely debunked.^48^

Sixth, this study did not take into account the presence and possible influence on TIII of non-TTLC tobacco companies such as local companies and state monopolies which also have the potential to interfere in tobacco control policy. We took this decision given the global dominance (Table 1) and power of the TTLCs.^8,16^

### Recommendations

First, countries should think carefully before accepting supply chain activity including foreign direct investment and the apparatus of supply chain activity from the TTLCs as it may compromise their ability to protect population health and the environment. Second, while high-income countries, as well as low-income countries, can be negatively impacted by hosting the supply chain, corporations’ financial power is large especially compared with the gross domestic product of LMICs. Supply chain activities take place in LMICs—they provide cheap labor and less regulation and geographical distance from high-income countries may make the bad practices of these companies easier to hide or ignore.^21,49,50^ High-income countries and international bodies should pay attention to the public health consequences of business activity in LMICs. Third, to better understand the implications of supply chain activities it would be useful for countries to require TTLCs to regularly provide detailed, up-to-date information about local supply chain activities in an accessible format. Fourth, the supply of tobacco has until now received little attention from tobacco control researchers and advocates^20^ and we recommend more work in this area to add to our understanding of the relationships between upstream supply chain activity and the undermining of public health.

## Conclusion

This paper shows the TTLCs’ dominance over the world’s tobacco supply chains in at least two ways. First, analysts rate them high on measures of company size. Second, the tobacco supply chain database reveals their subsidiaries are truly global. Even where governmental reporting requirements exist, TTLCs’ subsidiaries and their activities are not easy to track or understand. Importantly, our findings indicate that countries with fewer TTLC subsidiaries growing tobacco and fewer TTLC subsidiaries manufacturing tobacco products have less tobacco industry interference. We recommend that all countries require TTLCs to provide details of all tobacco-related activities carried out in their countries in an easy-to-process format to ease monitoring and ultimately encourage the decline of the production of tobacco products.

## Supplementary Material

A Contributorship Form detailing each author’s specific involvement with this content, as well as any supplementary data, are available online at https://academic.oup.com/ntr.

ntad178_suppl_Supplementary_Material

## Data Availability

The supply chains data collated are publicly available from the Tobacco Supply Chains Database.^23^ The TIII, country income group, MPOWER, World governance indicators and Comtrade exports are publicly available to download.^24–28^
