# Supplementary material for: Hosting the Tobacco Industry Supply Chain and Political Interference
Source: Nicotine Tob Res. 2023 Sep 14;25(12):1847–55. doi: 10.1093/ntr/ntad178 (PMC10664082; doi:10.1093/ntr/ntad178)
Supplement: ntad178_suppl_Supplementary_Material [file ntad178_suppl_supplementary_material.docx]

# **Hosting the tobacco industry supply chain and political interference – supplementary material**

Table S1 Tests of normality for bivariable analysis

|  | N TTLC growing | KS^1^ | df | Sig. | SW^2^ | df | Sig. | N TTLC  manufac | KS | df | Sig. | SW | df | Sig. | Country income | KS | df | Sig. | SW | df | Sig. |
| --- | --- | --- | --- | --- | --- | --- | --- | --- | --- | --- | --- | --- | --- | --- | --- | --- | --- | --- | --- | --- | --- |
| Interference | 0 | .053 | 43 | .200^*^ | .993 | 43 | .995 | 0 | .088 | 29 | .200^*^ | .978 | 29 | .790 | Low&low/mid | .095 | 32 | .200^*^ | .980 | 32 | .809 |
|  | 1 | .133 | 16 | .200^*^ | .966 | 16 | .774 | 1 | .130 | 18 | .200^*^ | .984 | 18 | .979 | Upper mid | .100 | 24 | .200^*^ | .978 | 24 | .862 |
|  | >1 | .209 | 17 | .047 | .945 | 17 | .387 | 2 | .174 | 19 | .131 | .948 | 19 | .372 | High | .143 | 20 | .200^*^ | .971 | 20 | .778 |
|  | **TOTAL** | **.081** | **76** | **.200^*^** | **.992** | **76** | **.914** | >2 | .182 | 10 | .200^*^ | .931 | 10 | .461 |  |  |  |  |  |  |  |
|  |  |  |  |  |  |  |  |  |  |  |  |  |  |  |  |  |  |  |  |  |  |
| MPOWER (index) | 0 | .124 | 43 | .093 | .945 | 43 | .041 | 0 | .130 | 29 | .200^*^ | .929 | 29 | .052 | Low&low/mid | .162 | 32 | .033 | .930 | 32 | .039 |
|  | 1 | .163 | 16 | .200^*^ | .923 | 16 | .190 | 1 | .142 | 18 | .200^*^ | .952 | 18 | .459 | Upper mid | .070 | 24 | .200^*^ | .988 | 24 | .988 |
|  | >1 | .139 | 17 | .200^*^ | .964 | 17 | .703 | 2 | .160 | 19 | .200^*^ | .914 | 19 | .089 | High | .227 | 20 | .008 | .879 | 20 | .017 |
|  | **TOTAL** | **.108** | **76** | **.029** | **.969** | **76** | **.060** | >2 | .249 | 10 | .078 | .867 | 10 | .093 |  |  |  |  |  |  |  |
|  |  |  |  |  |  |  |  |  |  |  |  |  |  |  |  |  |  |  |  |  |  |
| Monitor prevalence | 0 | .342 | 43 | <.001 | .745 | 43 | <.001 | 0 | .323 | 29 | <.001 | .742 | 29 | <.001 | Low&low/mid | .239 | 32 | <.001 | .830 | 32 | <.001 |
|  | 1 | .271 | 16 | .003 | .818 | 16 | .005 | 1 | .252 | 18 | .004 | .815 | 18 | .002 | Upper mid | .318 | 24 | <.001 | .725 | 24 | <.001 |
|  | >1 | .403 | 17 | <.001 | .635 | 17 | <.001 | 2 | .350 | 19 | <.001 | .742 | 19 | <.001 | High | .527 | 20 | <.001 | .351 | 20 | <.001 |
|  | **TOTAL** | **.344** | **76** | **<.001** | **.741** | **76** | **<.001** | >2 | .524 | 10 | <.001 | .366 | 10 | <.001 |  |  |  |  |  |  |  |
|  |  |  |  |  |  |  |  |  |  |  |  |  |  |  |  |  |  |  |  |  |  |
| Warnings on packs | 0 | .378 | 43 | <.001 | .691 | 43 | <.001 | 0 | .348 | 29 | <.001 | .728 | 29 | <.001 | Low&low/mid | .346 | 32 | <.001 | .694 | 32 | <.001 |
|  | 1 | .322 | 16 | <.001 | .729 | 16 | <.001 | 1 | .376 | 18 | <.001 | .702 | 18 | <.001 | Upper mid | .365 | 24 | <.001 | .717 | 24 | <.001 |
|  | >1 | .354 | 17 | <.001 | .714 | 17 | <.001 | 2 | .308 | 19 | <.001 | .753 | 19 | <.001 | High | .394 | 20 | <.001 | .675 | 20 | <.001 |
|  | **TOTAL** | **.362** | **76** | **<.001** | **.702** | **76** | **<.001** | >2 | .482 | 10 | <.001 | .509 | 10 | <.001 |  |  |  |  |  |  |  |
|  |  |  |  |  |  |  |  |  |  |  |  |  |  |  |  |  |  |  |  |  |  |
| Enforce ad bans | 0 | .403 | 43 | <.001 | .691 | 43 | <.001 | 0 | .377 | 29 | <.001 | .720 | 29 | <.001 | Low&low/mid | .375 | 32 | <.001 | .714 | 32 | <.001 |
|  | 1 | .368 | 16 | <.001 | .678 | 16 | <.001 | 1 | .465 | 18 | <.001 | .594 | 18 | <.001 | Upper mid | .386 | 24 | <.001 | .726 | 24 | <.001 |
|  | >1 | .320 | 17 | <.001 | .778 | 17 | .001 | 2 | .364 | 19 | <.001 | .743 | 19 | <.001 | High | .406 | 20 | <.001 | .709 | 20 | <.001 |
|  | **TOTAL** | **.391** | **76** | **<.001** | **.718** | **76** | **<.001** | >2 | .333 | 10 | .002 | .793 | 10 | .012 |  |  |  |  |  |  |  |
|  |  |  |  |  |  |  |  |  |  |  |  |  |  |  |  |  |  |  |  |  |  |
| Regulate (est) | 0 | .112 | 43 | .200^*^ | .966 | 43 | .221 | 0 | .105 | 29 | .200^*^ | .966 | 29 | .455 | Low&low/mid | .084 | 32 | .200^*^ | .981 | 32 | .839 |
|  | 1 | .163 | 16 | .200^*^ | .881 | 16 | .040 | 1 | .254 | 18 | .003 | .830 | 18 | .004 | Upper mid | .112 | 24 | .200^*^ | .978 | 24 | .858 |
|  | >1 | .208 | 17 | .049 | .862 | 17 | .017 | 2 | .144 | 19 | .200^*^ | .941 | 19 | .277 | High | .118 | 20 | .200^*^ | .951 | 20 | .387 |
|  | **TOTAL** | **.107** | **76** | **.030** | **.954** | **76** | **.008** | >2 | .259 | 10 | .056 | .829 | 10 | .032 |  |  |  |  |  |  |  |
|  |  |  |  |  |  |  |  |  |  |  |  |  |  |  |  |  |  |  |  |  |  |
| Regulate (rank) | 0 | .105 | 43 | .200^*^ | .956 | 43 | .103 | 0 | .077 | 29 | .200^*^ | .969 | 29 | .545 | Low&low/mid | .073 | 32 | .200^*^ | .986 | 32 | .945 |
|  | 1 | .150 | 16 | .200^*^ | .908 | 16 | .107 | 1 | .215 | 18 | .027 | .872 | 18 | .019 | Upper mid | .111 | 24 | .200^*^ | .967 | 24 | .594 |
|  | >1 | .167 | 17 | .200^*^ | .908 | 17 | .093 | 2 | .129 | 19 | .200^*^ | .934 | 19 | .207 | High | .131 | 20 | .200^*^ | .933 | 20 | .177 |
|  | **TOTAL** | **.077** | **76** | **.200^*^** | **.961** | **76** | **.020** | >2 | .202 | 10 | .200^*^ | .866 | 10 | .089 |  |  |  |  |  |  |  |
|  |  |  |  |  |  |  |  |  |  |  |  |  |  |  |  |  |  |  |  |  |  |
| Exports US$ | 0 | .352 | 43 | <.001 | .561 | 43 | <.001 | 0 | .333 | 29 | <.001 | .453 | 29 | <.001 | Low&low/mid | .276 | 32 | <.001 | .618 | 32 | <.001 |
|  | 1 | .427 | 16 | <.001 | .386 | 16 | <.001 | 1 | .374 | 18 | <.001 | .613 | 18 | <.001 | Upper mid | .356 | 24 | <.001 | .640 | 24 | <.001 |
|  | >1 | .327 | 17 | <.001 | .755 | 17 | .001 | 2 | .351 | 19 | <.001 | .681 | 19 | <.001 | High | .259 | 20 | .001 | .685 | 20 | <.001 |
|  | **TOTAL** | **.317** | **76** | **<.001** | **.530** | **76** | **<.001** | >2 | .247 | 10 | .084 | .805 | 10 | .016 |  |  |  |  |  |  |  |
|  |  |  |  |  |  |  |  |  |  |  |  |  |  |  |  |  |  |  |  |  |  |
| % exports tobacco | 0 | .349 | 43 | <.001 | .407 | 43 | <.001 | 0 | .338 | 29 | <.001 | .434 | 29 | <.001 | Low&low/mid | .264 | 32 | <.001 | .688 | 32 | <.001 |
|  | 1 | .275 | 16 | .002 | .717 | 16 | <.001 | 1 | .304 | 18 | <.001 | .704 | 18 | <.001 | Upper mid | .348 | 24 | <.001 | .411 | 24 | <.001 |
|  | >1 | .237 | 17 | .012 | .704 | 17 | <.001 | 2 | .407 | 19 | <.001 | .419 | 19 | <.001 | High | .287 | 20 | <.001 | .559 | 20 | <.001 |
|  | **TOTAL** | **.310** | **76** | **<.001** | **.513** | **76** | **<.001** | >2 | .252 | 10 | .071 | .859 | 10 | .073 |  |  |  |  |  |  |  |

^1^Kolmogorov-Smirnov with Lilliefors significance correction ^2^Shapiro-Wilk *lower bound of true significance

Table S2 Bivariable analysis

|  | Tobacco Industry  interference | | N TTLC undertaking tobacco growing | | | | | N TTC undertaking final product manufacturing | | | | | |
| --- | --- | --- | --- | --- | --- | --- | --- | --- | --- | --- | --- | --- | --- |
|  | rho | p | 0 | 1 | >1 | Total | p | 0 | 1 | 2 | >2 | Total | p |
| N |  |  | 43 | 16 | 17 | 76 |  | 29 | 18 | 19 | 10 | 76 |  |
|  | Correlation | | Median | | | |  | Median | | | | |  |
| Interference (TIII) | 1 |  | 56 | 53 | 72 | 57 | .004 | 50 | 60 | 55 | 66 | 57 | .033 |
|  |  |  |  |  |  |  |  |  |  |  |  |  |  |
| Tobacco Control |  |  |  |  |  |  |  |  |  |  |  |  |  |
| MPOWER (index) | -.281 | .014 | 4.2 | 3.9 | 4.0 | 4.2 | .759 | 4. 3 | 3.4 | 4.0 | 4.2 | 4.2 | .444 |
| Monitor (prevalence data) (score) | -.073 | .533 | 3 | 2 | 3 | 3 | .488 | 3 | 2 | 3 | 3 | 3 | .085 |
| Warnings on packs (score) | -.219 | .057 | 3 | 3 | 3 | 3 | .899 | 3 | 3 | 3 | 3 | 3 | .417 |
| Enforce advert bans (score) | -.271 | .018 | 2 | 2 | 2 | 2 | .226 | 2 | 2 | 2 | 2 | 2 | .543 |
|  |  |  |  |  |  |  |  |  |  |  |  |  |  |
| World governance |  |  |  |  |  |  |  |  |  |  |  |  |  |
| Voice (est) | -.020 | .865 | .100 | -.489 | -.074 | -.023 | .175 | .015 | -.258 | -.637 | .217 | -.023 | .493 |
| Voice (rank) | -.020 | .865 | 51 | 34 | 44 | 46 | .175 | 47 | 38 | 30 | 54 | 46 | .493 |
| Stability (est) | -.051 | .662 | -.003 | -.506 | -.437 | -.249 | .125 | -.211 | -.129 | -.254 | -.496 | -.249 | .623 |
| Stability (rank) | -.051 | .662 | 47 | 29 | 31 | 38 | .125 | 41 | 42 | 38 | 28 | 38 | .623 |
| Govt effective (est) | -.060 | .604 | .031 | -.270 | -.087 | -.085 | .428 | -.118 | -.381 | .031 | -.008 | -.085 | .717 |
| Govt effective (rank) | -.060 | .604 | 55 | 41 | 50 | 50 | .428 | 48 | 37 | 55 | 54 | 50 | .717 |
| Regulate private sector (est) | -.063 | .590 | .090 | -.461 | -.111 | -.096 | .078 | .083 | -.408 | -.312 | .187 | -.096 | .207 |
| Regulate private sector (rank) | -.063 | .590 | 56 | 36 | 48 | 49 | .078 | 55 | 38 | 42 | 59 | 49 | .207 |
| Rule of law (est) | -.117 | .313 | -.096 | -.346 | -.416 | -.246 | .427 | -.225 | -.298 | -.364 | -.253 | -.246 | .974 |
| Rule of law (rank) | -.117 | .313 | 50 | 41 | 37 | 45 | .427 | 46 | 43 | 40 | 44 | 45 | .974 |
| Control corruption (est) | -.104 | .372 | -.059 | -.515 | -.401 | -.350 | .202 | -.457 | -.238 | -.286 | -.407 | -.350 | .953 |
| Control corruption (rank) | -.104 | .372 | 52 | 33 | 39 | 43 | .202 | 35 | 48 | 47 | 39 | 43 | .953 |
|  |  |  |  |  |  |  |  |  |  |  |  |  |  |
| Tobacco contribution to economy |  |  |  |  |  |  |  |  |  |  |  |  |  |
| Tobacco exports (US$ Millions) | .336 | .003 | 39 | 83 | 206 | 76 | .002 | 18 | 103 | 167 | 1,030 | 76 | <.001 |
| % total export value is tobacco | .336 | .005 | .1 | .5 | .4 | .2 | .020 | .1 | .5 | .2 | .7 | .2 | .021 |

Box S1 Tests of suitability of TIII for ANOVA and generalised linear modelling dependent variable


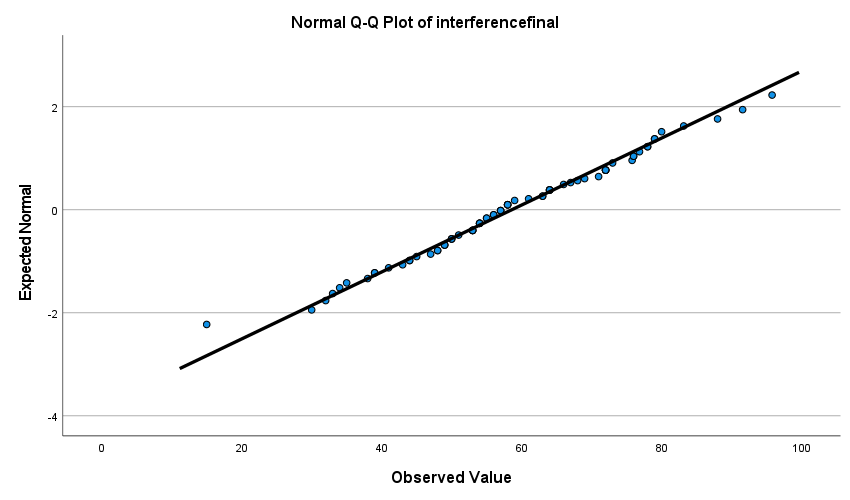

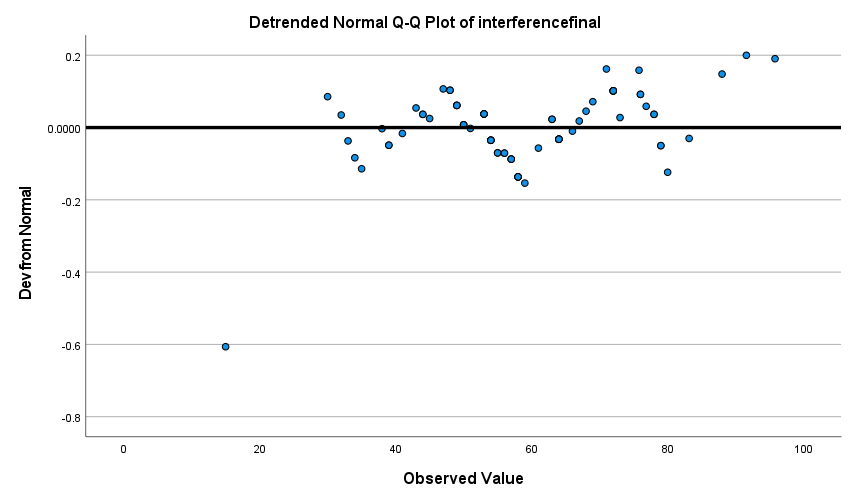


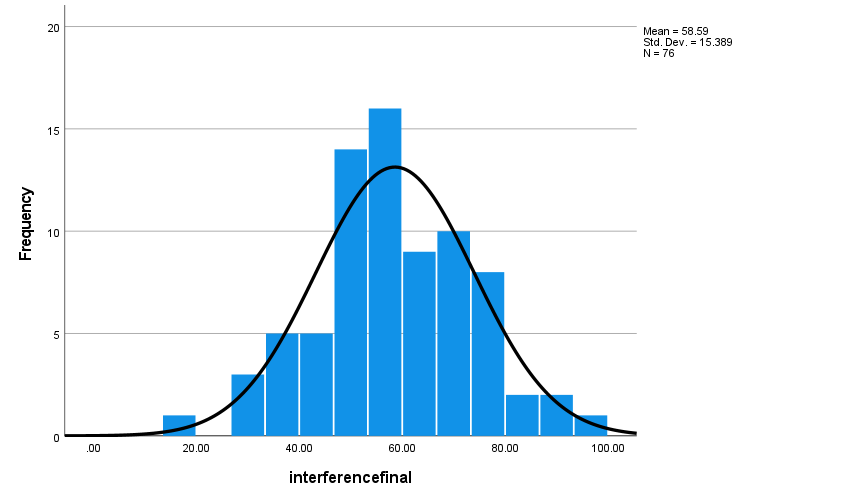


Tests of homogeneity of variance (Levine Statistic)

|  | N TTLC  growing | df1 | df2 | Sig. |  | N TTC making  final product | df1 | df2 | Sig. |  | Country  income | df1 | df2 | Sig. |
| --- | --- | --- | --- | --- | --- | --- | --- | --- | --- | --- | --- | --- | --- | --- |
| Based on Mean | 2.392 | 2 | 73 | .099 |  | .125 | 3 | 72 | .945 |  | 2.843 | 2 | 73 | .065 |
| Based on Median | 2.365 | 2 | 73 | .101 |  | .085 | 3 | 72 | .968 |  | 2.800 | 2 | 73 | .067 |
| Based on Median and with adjusted df | 2.365 | 2 | 66.1 | .102 |  | .085 | 3 | 68.3 | .968 |  | 2.800 | 2 | 54. 4 | .070 |
| Based on trimmed mean | 2.388 | 2 | 73 | .099 |  | .126 | 3 | 72 | .944 |  | 2.842 | 2 | 73 | .065 |

Table S3 Building the base model: bivariable and multivariable associations between country income, subsidiaries, and interference

|  |  | ANOVA | Bivariable regression | | | Multivariable regression | | | | | | | | | | | |
| --- | --- | --- | --- | --- | --- | --- | --- | --- | --- | --- | --- | --- | --- | --- | --- | --- | --- |
|  |  |  |  |  |  | Independent variables: Income + growing | | | Independent variables:  Income + final product manufacture | | | **FINAL MODEL**  **Independent variables: Income+ growing + final product manufacture** | | | *Stress test – final model without outlier Brunei Darussalam (N=75)* | | |
|  | N | Mean (95%CI) | B | SE | p | B | SE | p | B | SE | p | **B** | **SE** | **p** | *B* | *SE* | *p* |
|  |  |  |  |  |  |  |  |  |  |  |  |  |  |  |  |  |  |
| *Total* | 76 | 58.6 (55.1 to 62.1) |  |  |  |  |  |  |  |  |  |  |  |  |  |  |  |
|  |  |  |  |  |  |  |  |  |  |  |  |  |  |  |  |  |  |
| *Country income* |  | P=.006 |  |  | .004 |  |  | .003 |  |  | <.001 |  |  | **<.001** |  |  | *<.001* |
| Lower middle (&low) income | 32 | 56.1 (51.8 to 60.4) | 3.0 | 4.1 | .464 | 3.2 | 3.9 | .412 | 2.4 | 3.7 | .515 | **3.0** | **3.6** | **.412** | *1.4* | *3.5* | *.681* |
| Upper middle income | 24 | 66.5 (61.2 to 71.7) | 13.4 | 4.3 | .002 | 12.7 | 4.0 | .002 | 15.6 | 4.0 | <.001 | **15.0** | **3.8** | **<.001** | *13.1* | *3.8* | *<.001* |
| High income* | 20 | 53.1 (43.8 to 62.5) | 0 |  |  | 0 |  |  |  |  |  | **0** |  |  | *0* |  |  |
|  |  |  |  |  |  |  |  |  |  |  |  |  |  |  |  |  |  |
|  |  |  |  |  |  |  |  |  |  |  |  |  |  |  |  |  |  |
| *N TTLC tobacco growing* |  | P=.007 |  |  | .004 |  |  | .004 |  |  |  |  |  | **.046** |  |  | *.031* |
| no TTLCs | 43 | 56.8 (51.6 to 61.9) | -11.6 | 4.1 | .004 | -11.4 | 3.8 | .003 |  |  |  | **-6.8** | **4.0** | **.085** | *-6.8* | *3.8* | *.077* |
| 1 TTLC | 16 | 53.1 (47.5 to 58.7) | -15.3 | 5.0 | .002 | -13.8 | 4.7 | .003 |  |  |  | **-11.5** | **4.7** | **.014** | *-11.8* | *4.5* | *.008* |
| >1 TTLCs * | 17 | 68.4 (62.4 to 74.4) | 0 |  |  | 0 |  |  |  |  |  | **0** |  |  | *0* |  |  |
|  |  |  |  |  |  |  |  |  |  |  |  |  |  |  |  |  |  |
|  |  |  |  |  |  |  |  |  |  |  |  |  |  |  |  |  |  |
| *N TTC final product manufacturing* |  | P=.030 |  |  | .018 |  |  |  |  |  | .001 |  |  | **.008** |  |  | *.016* |
| no TTCs | 29 | 52.7 (46.9 to 58.6) | -15.4 | 5.3 | .003 |  |  |  | -16.2 | 4.7 | .001 | **-12.7** | **4.9** | **.010** | *-11.4* | *4.7* | *.016* |
| 1 TTC | 18 | 60.6 (53.4 to 67.8) | -7.6 | 5.7 | .182 |  |  |  | -3.8 | 5.2 | .464 | **-0.5** | **5.2** | **.923** | *-0.5* | *5.0* | *.921* |
| 2 TTCs | 19 | 60.6 (53.4 to 67.8) | -7.6 | 5.6 | .177 |  |  |  | -5.9 | 5.1 | .247 | **-5.2** | **4.9** | **.285** | *-5.2* | *4.7* | *.268* |
| >2 TTCs * | 10 | 68.2 (58.8 to 77.5) | 0 |  |  |  |  |  | 0 |  |  | **0** |  |  | *0* |  |  |
|  |  |  |  |  |  |  |  |  |  |  |  |  |  |  |  |  |  |
|  |  |  |  |  |  |  |  |  |  |  |  |  |  |  |  |  |  |
| Model Fit (Log Likelihood) | | | | | | | | | | | | | | | | | |
|  |  |  |  |  |  |  |  |  |  |  |  |  |  |  |  |  |  |
| Income |  |  | -309.840 | | |  |  |  |  |  |  |  |  |  |  |  |  |
| *N TTLC tobacco growing* |  |  | -309.924 | | |  |  |  |  |  |  |  |  |  |  |  |  |
| *N TTC final product manufacturing* |  |  | -310.392 | | |  |  |  |  |  |  |  |  |  |  |  |  |
|  |  |  |  |  |  |  |  | - |  |  |  |  |  |  |  |  |  |
| Model |  |  |  |  |  | -304.636 | | | -302.041 | | | **-299.087** | | | *-.292.226* | | |

*Reference category: in multivariable modelling, interference scores of the other within-variable groupings are compared to the reference category
